# Oxygen Reduction Reaction on N-Doped Graphene: Effect of Positions and Scaling Relations of Adsorption Energies

**DOI:** 10.1021/acs.jpcc.0c11340

**Published:** 2021-04-20

**Authors:** Ádám Ganyecz, Mihály Kállay

**Affiliations:** Department of Physical Chemistry and Materials Science, Budapest University of Technology and Economics, P.O.Box 91, Budapest H-1521, Hungary

## Abstract

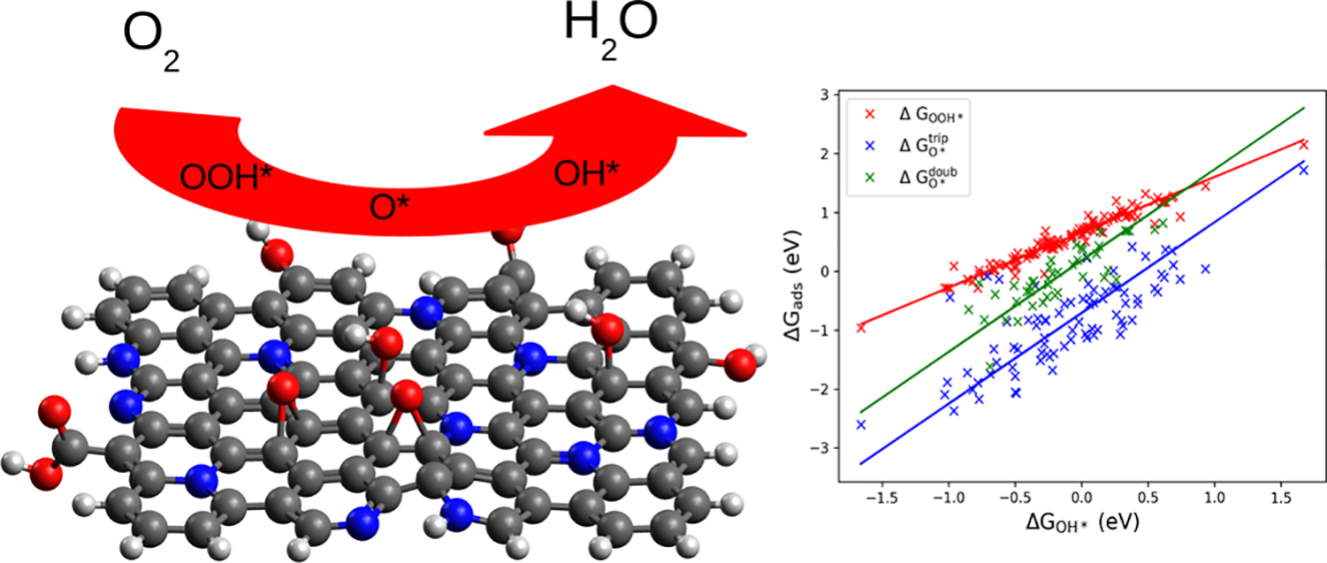

The goal of this study is to provide insight into the mechanism of the oxygen reduction reaction (ORR) on N-doped graphene surfaces. Using density functional theory and a computational hydrogen electrode model, we studied the energetics of the ORR intermediates, the effect of the position of the reaction site, and the effect of the position of the N modification relative to the active site on model graphene surfaces containing one or two N atoms. We found that scaling relations can be derived for N-doped graphenes as well, but the multiplicity of the surface should be taken into account. On the basis of the scaling relations between intermediates OOH* and OH*, the minimal overpotential is 0.33 V. Analysis of the data showed that N atoms in the meta position usually decrease the adsorption energy, but those in the ortho position aid the adsorption. The outer position on the zigzag edge of the graphene sheet also promotes the adsorption of oxygenated species, while the inner position hinders it. Looking at the most effective active sites, our analysis shows that the minimal overpotential can be approached with various doping arrangements, which also explains the contradicting results in the literature. The dissociative pathway was also investigated, but we found only one possible active site; therefore, this pathway is not really viable. However, routes not preferred thermodynamically pose the possibility of breaking the theoretical limit of the overpotential of the associative pathway.

## Introduction

Polymer electrolyte fuel cells (PEFC) are widely investigated because they are promising alternatives to petroleum-based energy sources due to their high efficiency, high power density, low emission, and low operating temperature.^1^ However, there are still many obstacles to tackle before PEFCs can be applicable. One of them is to find a sufficiently active and cheap catalyst for the oxygen reduction reaction (ORR). Currently, Pt is used as a cathode catalyst, but its high cost and unsatisfactory activity prevent its commercialization.

In the past decade, graphene and especially N-doped graphene are widely researched materials as catalysts for the ORR. Several preparation methods have been developed to synthesize N-doped carbons, and they were proved to be as efficient catalysts as Pt/C if not more effective and durable.^2−17^ However, regarding the mechanism and active center, there are contradictions. The two main types of nitrogens distinguished by experimentalists are the N’s simply replacing a carbon atom in the bulk graphene sheet, called graphitic-N’s, and the N’s replacing a carbon atom at the edge of the sheet, referred to as pyridinic-N’s. Usually the graphitic, pyridinic, and other N content is determined with X-ray photoelectron spectroscopy, and the electrocatalytic effect is investigated depending on the different N content. On the basis of their results, several papers have suggested that the pyridinic N is the main contributor to the ORR activity,^18−28^ while others suspect that the C atom’s neighboring graphitic N’s are the active center for the ORR.^29−35^ According to a third standpoint, both types of N’s have their own role. Lai et al.^4^ prepared two types of N-doped graphenes by annealing and found that graphitic N’s increase the limiting current density and that pyridinic N’s improve the onset potential. Qiao and associates^7^ fabricated N-doped carbon/graphene–oxide composites. They claim that the material should reach a certain conductivity to access all active sites and to become the main contributor of the ORR activity. He and co-workers^16^ used ketoamine condensation reactions to create N-doped graphene nanoplatelets with pyrrolic N, pyridinic N, and pyridinic N-oxide. They found that although the improvement of the ORR performance benefits from N doping, the edge-selective nitrogen doping does not play an important role in the enhancement of the ORR activity in alkaline media.

Various quantum chemical methods have also been used to resolve the mechanism of the ORR and find the active center on graphene and N-modified graphenes. However, the results of these studies are also contradictory. Most of the authors considered only the pure or modified graphene sheet, while no attention was paid to the edges. It was found that the ORR can produce a significant amount of H_2_O_2_ using methods like density functional theory (DFT),^36^ periodic DFT,^37^ and molecular dynamics (MD).^38^ However, the periodic DFT calculation of Yu et al. showed that the formation of H_2_O_2_ in alkaline solutions is unfavorable.^39^ There are several publications that consider the edges of graphene sheets as well, but the zigzag (ZZ) and armchair (AC) edges are usually not studied separately.^40−42^ In the combined study of Jiao and co-workers,^42^ it was found that there is good agreement between the experimental data and the DFT calculations concerning the ORR performance of B-, N-, O-, S-, and P-doped graphene in alkaline solution. Their results suggest that the most effective adsorption sites are near graphitic N’s on N-doped graphene, while the edges are less reactive, but the ZZ and AC edges were not distinguished in this study. However, in other works^43−45^ where the edges were separately studied, the ZZ edge near the substituted N atom showed the highest catalytic effect. Also, there are signs that the AC edge with N has a better catalytic effect.^46^ Besides the above investigations, the effect of the number of N atoms in the graphene sheet on the ORR is not a well-studied field. In a single study, Zhang and co-workers^41^ found that more than two nitrogen atoms reduce the number of active sites, while the introduction of defects enhances the catalytic effect.

Our goal in this study is to explore the effect of N atoms on the energetics of the the ORR on N-doped graphene taking into account all possible associative and dissociative pathways. We study in detail how the different positions of the doping N atoms affect the catalytic activity. Also, we try to resolve the contradictions in the literature.

The article is structured as follows. After summarizing the computational details, we investigate the associative pathway of the ORR including (a) the first steps of the ORR, (b) the relationships of the energies of the key intermediates of the ORR, and (c) the effect of the positions of N’s on the ORR activity. Then, we discuss the energetics of the dissociative pathway. Finally, we also consider the applicability of the results from a practical point of view.

## Methods

For the geometry optimizations and frequency calculations, the B3LYP functional^47^ was applied with the 6-31G** basis set^48^ in combination with the DFT-D3 dispersion correction^49^ with Becke–Johnson damping.^50^ The SMD solvation model^51^ was employed to model solvent effects. The computational hydrogen electrode model developed by Nørskov et al.^52^ was utilized to calculate the free energies of the ORR intermediates, which has been applied in several studies.^53−57^ In this approach, using the standard hydrogen electrode (SHE) as a reference, the chemical potential of H^+^ and e^–^ can be related to that of 1/2 H_2_ in the gas phase, i.e., at *U* = 0 V, pH = 0, *p* = 1 bar, and *T* = 298 K, *G*(H^+^) + *G*(e^–^) = 1/2 *G*(H_2_). The Gibbs free energy of a species at 298 K, *G*_298_, was calculated as
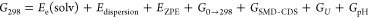
1where *E*_e_(solv) is the relative electronic energy calculated at the B3LYP/6-31G** level using the SMD solvation model, while *E*_dispersion_ is the corresponding dispersion correction. *E*_ZPE_ and *G*_0→298_ denote the differences of the zero-point energies and the thermal corrections to the Gibbs free energies, respectively, also obtained from B3LYP/6-31G** calculations. *G*_SMD–CDS_ stands for the nonelectrostatic effect of solvation from the SMD solvation model. *G*_*U*_ incorporates the effect of the electrode potential (*U*), which is equal to −*eU* with *e* as the elementary charge. This means that the energies can be easily transformed to any *U* voltage by adding −*neU* to the Gibbs free energy, where *n* is the number of electrons present in that state. *G*_pH_ is the change in free energy due to the presence of H^+^ ions in the solution, the activity of which is, however, taken as unity, so this term has no effect on the results. Since the high-spin ground state of the oxygen molecule is described unreliably in DFT calculations,^58^ the free energy of O_2_(g) was determined as *G*(O_2_(g)) = 2*G*(H_2_O(l)) – 2*G*(H_2_(g)) – 4.92 eV. The quantum chemical calculations were carried out with the Gaussian 09 quantum chemistry program (Revision E.01).^59^

**Figure 1:**
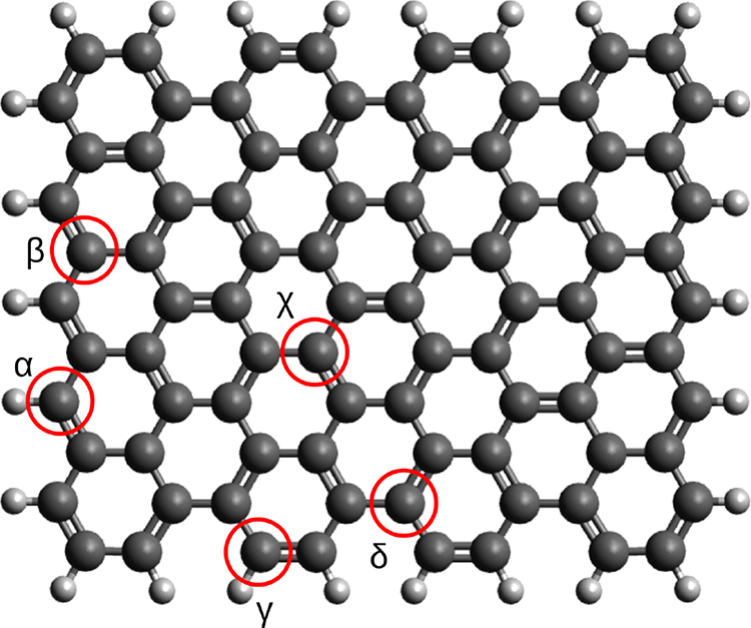
Base graphene sheet model and explanation of the notation used in this work.

**Figure 2:**
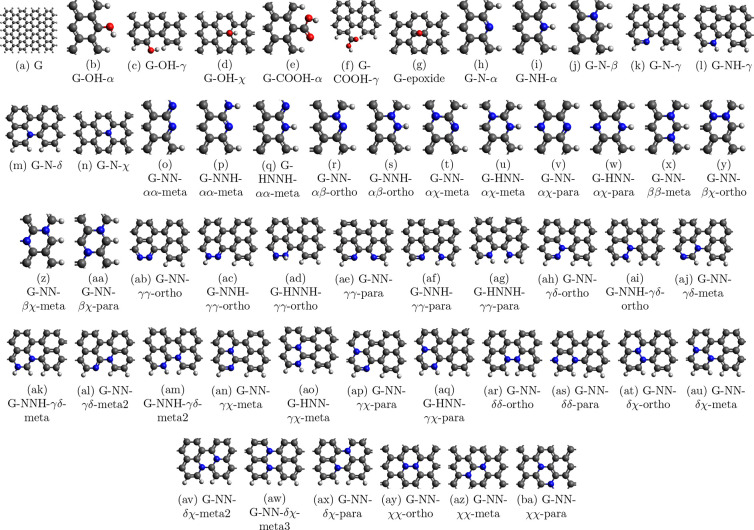
All investigated graphene surfaces. (b)–(ba) have the same same dimensions as (a), but only the relevant parts are highlighted.

Figure 1 presents the base graphene used in this work with the notations of the positions. Figure 2 shows all of the various graphene surfaces studied based on the base graphene, highlighting the relevant part of the sheet. The graphene sheets are named in the following manner: G-“modification”-“absolute position of modification”-“relative position to each other”, for example, G-NN-*αχ*-para. Here, “modification” can be OH, COOH, epoxy groups, and N atoms; NN means that two N atoms are inserted in the graphene sheet. “Absolute position of modification” can take the value of α or β, which are the outer and inner atoms of the zigzag edge; γ or δ, which are the outer and inner atoms of the armchair edge; and χ, which refers to the position of any other atom on the graphene sheet. “Relative position to each other” means that the distance of the modification is 1 (ortho), 2 (meta), or 3 (para) bonds in a ring.

## Results and Discussion

### Oxygen Adsorption and the First Reduction Step

**Figure 3:**
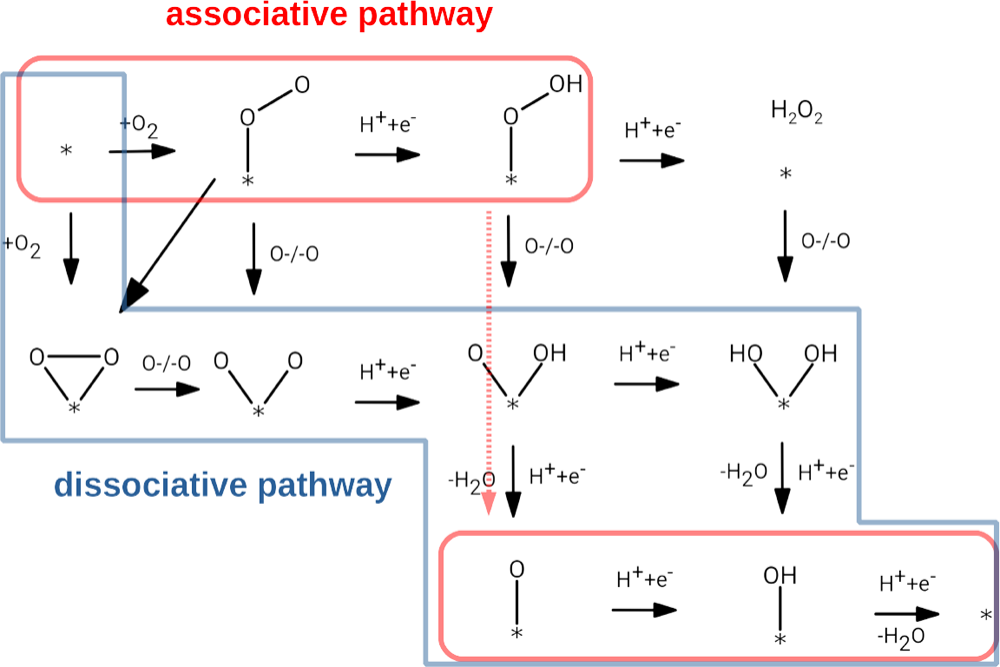
General mechanism of the ORR in acidic media. Asterisk denotes the surface of the catalyst. Red highlight shows the associative pathway and blue the dissociative pathway.

The general mechanism of the ORR in acidic media can be seen in Figure 3. The main mechanism of the ORR on graphene, the associative pathway (red route in Figure 3), goes through the OOH* intermediate; then it is further reduced to O* with simultaneous reduction and O–O bond breaking. In the last two reduction steps, we get OH*; finally, the clear surface is retrieved, and two water molecules form.

A dissociative pathway is also possible (blue route in Figure 3) where both O atoms are connected to a carbon atom after the O_2_ adsorption, then O–O bond breaking occurs, and both O atoms are reduced to H_2_O through OH* independently of each other.

If coupled proton and electron transfer is assumed, the first step is the formation of O_2_*, i.e., oxygen adsorption. However, in most cases, this process is not favored because the Gibbs free energy of the adsorption is positive, even though the formation of OOH* is thermodynamically preferred.

Several mechanisms were proposed in the literature (mostly for Pt) for the first reduction step. Yeager proposed a dissociative O_2_ adsorption pathway,^60−62^ while Damjanovich suggested that a proton and charge are simultaneously transferred to the weakly adsorbed O_2_.^63−65^ Wang and Balbuena presented a combined pathway:^66^ O_2_ + H^+^ → OOH^+^ and then OOH^+^ + * + e^–^ → OOH* or O_2_ + * + e^–^ → O_2_^–^* and then O_2_^–^* + H^+^ → OOH*.

To find out which pathway is more probable on graphene, we calculated the relative energies of both the O_2_^–^* and the HO_2_^+^* species on several surfaces, defined, respectively, by
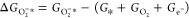
2
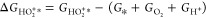
3

For a proper thermochemical analysis, the solvated H^+^ and e^–^ energies are also needed at 0 V vs SHE. On the basis of the work of Llano and Eriksson,^67^ the Gibbs free energy of H^+^ is −11.6511 eV, and the energy of e^–^ is −4.44 eV in the current setup. The relevant relative energies of the first step can be seen in Table 1 for a couple of selected surfaces. Here, only the surfaces with a single modification were considered as they are representative enough for this investigation. It is evident that OOH^+^* is more stable than O_2_^–^*; therefore, the combined oxygen and proton adsorption is the preferred mechanism for the formation of OOH*, i.e., * + O_2_ + e^–^ + H^+^ → HO_2_^+^* + e^–^ → OOH*. Also in almost every case, the combined adsorption is exergonic. It must be noted that in solute phase, the surface-independent process of HO_2_ formation then adsorption, that is, the O_2_ → O_2_^–^ → HO_2_ → HO_2_* route, cannot be ruled out.^68^

**Table 1:** Relative Gibbs Free Energies (in eV) of the Relevant Species for the Possible Mechanisms of the Formation of OOH* on Selected Surfaces

surface	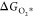	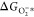	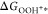	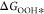
G-α	–0.42	0.40	–1.72	–1.52
G-γ	0.79	1.52	–0.45	0.18
G-OH-α-1	–0.41	–0.11	–1.69	–1.52
G-OH-γ-2	1.07	1.93	–0.13	0.16
G-OH-χ-1	1.51	1.82	–0.26	–0.05
G-N-α-1	–0.07	0.71	–1.40	–1.26
G-N-β-1	–0.63	0.50	–2.13	–1.21
G-N-γ-3	1.57	n.a.^a^	–0.17	0.36
G-N-δ-1	0.46	0.46	–1.60	–1.38
G-N-χ-1	0.75	1.05	–1.04	–0.76
G-NH-α	0.67	1.49	–0.91	–0.29
G-NH-γ-1	–0.13	0.65	–1.26	–1.04

a No adsorption; no bond formation between species and surface.

### Relationship between the Intermediates and the Overpotential

As we ascertained, the formation of OOH* is possible, even though the O_2_ adsorption is not preferred. Now we can study the main route of the ORR
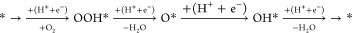
4In the following, instead of discussing each surface one by one, we try to get a more general picture about the effects and relationships regarding the ORR on doped graphene surfaces.

The key quantities which our proposition is based on are as follows. The relative Gibbs free energies of the key intermediate states can be written as
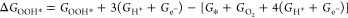
5
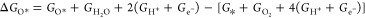
6
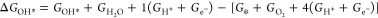
7whereas the energy requirements of the reduction steps can be calculated as
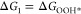
8
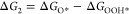
9
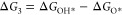
10
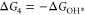
11

The overpotential (η) at 1.23 V vs SHE can be determined as
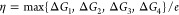
12Note that in the ideal case, at 1.23 V vs SHE, the overpotential is 0 V and the Gibbs free energy change of each step should be 0 eV.

**Figure 4:**
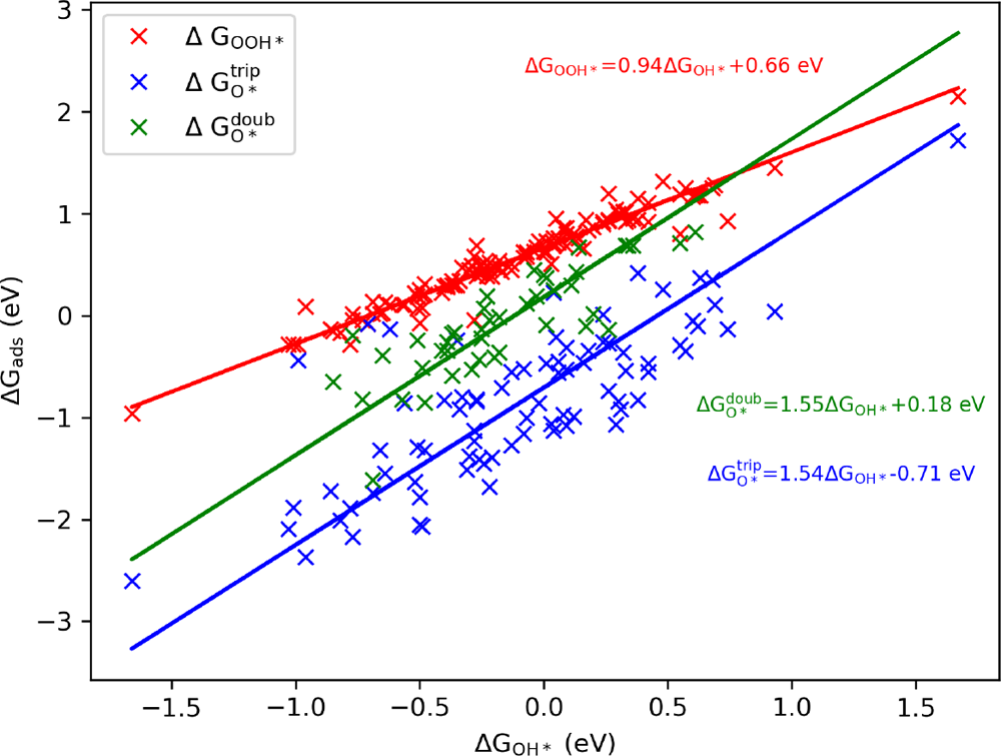
Relationship of the relative Gibbs free energies of the adsorbed species OOH* and O* with that of OH* at 1.23 V vs SHE. Δ*G*_ads_ = Δ*G*_OOH*_, Δ*G*_3_^trip^, and Δ*G*_O*_^doub^, where “trip” and “doub” refer to triplet and doublet states, respectively.

In Figure 4, the relation between the energies of OOH*, O*, and OOH* can be seen at 1.23 V applied voltage. A linear relation can be observed between the relative energies of OOH* and OH*; the equation of the fitted line is Δ*G*_OOH*_ = 0.94Δ*G*_OH*_ + 0.66 eV using the total least-squares method. This is in line with the previous findings for metals, metal–oxides,^69−73^ and graphene surfaces,^15,46,74^ where similar fitting parameters were found for this so-called scaling relation. The slope of almost 1.0 can be interpreted as the same interactions are playing a role in the adsorption of OH and OOH, and the bond order between O and the surface is the same in the two cases. If we consider that Δ*G*_OOH**_ = Δ*G*_1_ and – Δ*G*_OH*_ = Δ*G*_4_, supposing a slope of unity, the fitted linear equation can be rearranged as Δ*G*_1_ + Δ*G*_4_ = 0.66 eV. This represents a lower limit for the overpotential at 1.23 V applied voltage vs SHE. In the optimal case, Δ*G*_1_ = Δ*G*_4_ = 0.33 eV, i.e., η = 0.33 V, so it is not possible to obtain more than 0.9 V out of these batteries. This also shows the importance of the Sabatier principle, that is, the interactions between the catalyst and the substrate should be neither too strong nor too weak.

In contrast to metals and metal oxides, where the oxygen binds to the surface with a double bond, for graphene, the O adsorption cannot be related to the other species as precisely. The reason for this is that the oxygen can bind through various modes, mainly as epoxy-type or keto-type O. In addition, the keto-type bonding can be a proper double bond between the carbon and the oxygen, or as in most cases, it can make a bond with a bond order around 1.5 according to natural bond orbital (NBO) analysis.^75^ If we only plot the cases where the bond order is ∼1.5 and separate the data by the multiplicity of the graphene surface, we get two data sets and a line can be fitted on both with a slope around the bond order, ∼1.5–1.6. Basically, the slope of the scaling relation is the ratio of the bond orders for the adsorbed species, even though O* has an unusual bond order of 1.5–1.6. If we scale the Δ*G*_O*_ energies with the bond order, we should get a fitted linear equation with a slope of unity. However, when NBO bond orders are used, the slopes are 0.90 and 0.81 for the doublet and triplet surfaces, respectively. Looking at the localized orbitals of O*, it can be realized that the nonbonding p orbitals of O interact with the C atom, giving a small π bond character to the p orbitals, which explains the noninteger part of the bond order (see Figure S2 in the SI). In the cases of OOH* and OH*, this interaction does not exist; the nonbonding p orbitals of O are undisturbed. We also note that the adsorption energy of O can also depend on various further properties, like the tensile strength and the curvature of graphene,^76^ which are not considered here.

**Figure 5:**
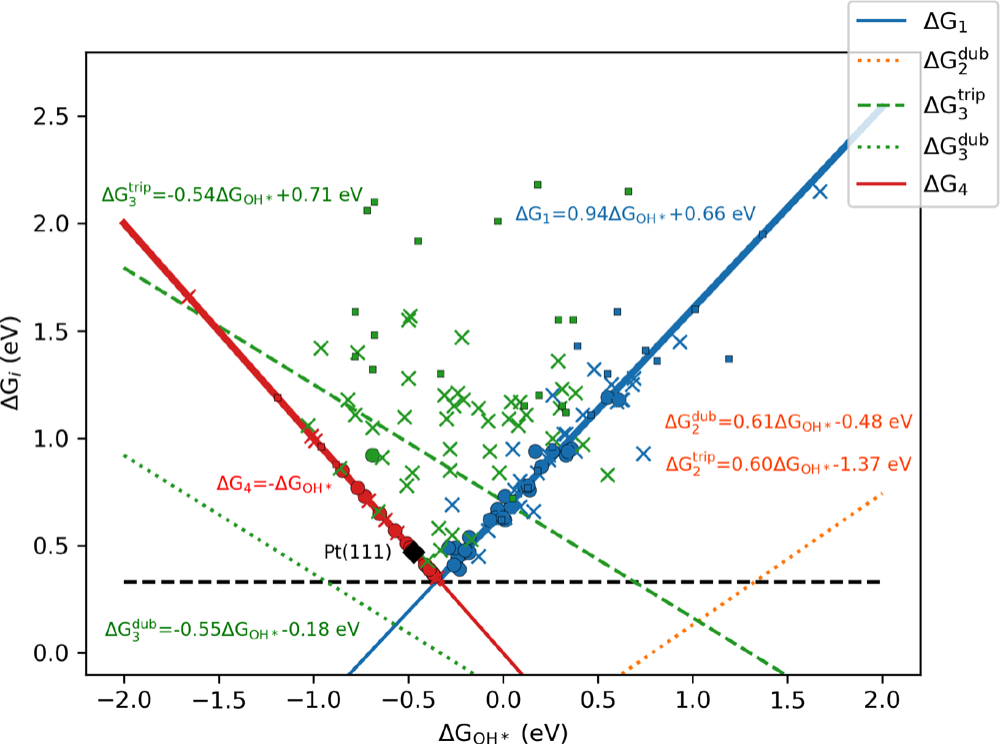
Gibbs free energy changes of the reduction steps as a function of Δ*G*_OH*_ at 1.23 V vs SHE. Dots and crosses represent the highest Gibbs free energy change for the studied reaction sites for triplet and doublet surfaces, respectively. Squares show the data for reaction sites which were left out from the regression due to their different O–* bond. Different colors are used for the various reduction steps (blue, Δ*G*_1_; orange, Δ*G*_2_; green, Δ*G*_3_; red, Δ*G*_4_). Dashed black line represents the theoretical minimum of the maximum of {Δ*G*_1_, Δ*G*_2_, Δ*G*_3_, and Δ*G*_4_}. Data for Pt(111) is taken from ref (77) and represented with a black diamond for comparison.

Furthermore, the fitted equations can be used to express the Δ*G*_*i*_ energy steps based on Δ*G*_OH*_. Figure 5 shows the resulting lines representing the energy changes of the four reduction steps together with the corresponding energy changes calculated for the reaction sites. For each reaction site, the rate-determining step is considered, and the corresponding data point is displayed accordingly. The sites where the first, second, third, and fourth steps require the largest change in the Gibbs free energy are represented by blue, orange, green, and red symbols, respectively, while dots and crosses refer to reaction sites where the rate-determining step is on the doublet and triplet surface, respectively. For a full picture, the reaction sites which were left out from the regression due to their different O–* bonding are also shown in Figure 5 with squares. Most of the data show that the overpotential is determined by the relationship between Δ*G*_1_ and Δ*G*_4_, especially for the doublet surfaces, but in several cases, the third step determines the overpotential due to the overbinding of O on the triplet surfaces. The higher energy requirement for Δ*G*_3_ on triplet surfaces (dashed green line) leads to a higher overpotential, which is obvious as the red and blue lines representing Δ*G*_1_ and Δ*G*_4_ are under the line of Δ*G*_3_^trip^ in the practically relevant region. Even if the fitted scaling relations for Δ*G*_O*_ against Δ*G*_OH*_ are not so accurate due to the variance in the C–O bond, the trends are clear. Consequently, not only the OOH and OH binding should be balanced, but also the strength of the O–* bond should be moderated.

The scaling relation for metals was previously described in the literature. It was found that Pt(111) is already close to the minimal overpotential^52,77^ with 0.47 V in this model. The deviation from the optimum is due to the strong binding of OOH, as it can be seen in Figure 5. It can be further improved in various ways, such as using Pt nanostructures^78^ or with cocatalysts.^79^ Our results simply show that fine tuning other materials, in this case N-doped graphene, could also lead to this minimal overpotential. However, it is still a challenge to create such active centers by doping, compared to Pt, which is already active on its whole surface.

### Effect of the Position on Graphene and the Position of N’s

Instead of discussing the catalytic effect of each surface and position one by one, we endeavor to give a more general description highlighting the main observations and conclusions. To that end, we elaborated a simple statistical model to quantify the effect of the topography of the surface on the rate of the ORR. Each site was labeled by its absolute position on the graphene sheet (α, β, γ, δ, and χ; see Figure 1), by the type and position of the N atom relative to the reaction site (ortho, meta, para), and by the multiplicity of the surface (doublet, triplet). Then, the effect of the positions and the types of N atoms on the adsorption energies were estimated by fitting the linear equation
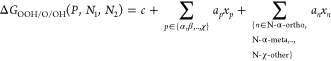
13where Δ*G*_OOH/O/OH_ is the relative Gibbs free energy of the species at 1.23 V vs SHE as a function of the position of the active site (*P*) and the type and position of the surrounding N’s (*N*_1_, *N*_2_). The *N*_1_ and *N*_2_ variables can take the values of N-α, N-β, N-γ, N-δ, and N-χ which refer to the N’s at the α, β, γ, δ, and χ. positions, respectively. The N’s can be hydrogenated in the α and γ positions, and these are denoted by NH-α and NH-γ, respectively. The position of the N atom can be ortho, meta, para, or other relative to the active site. *a*_*p*_ is the effect of the position of the reaction site *p* on the adsorption energy; *x*_*p*_ is 1 if *P* = *p* and 0 otherwise. *a*_*n*_ is the effect of the N atoms considering their type and position, and *x*_*n*_ is the number of that particular type of N’s in the corresponding position. Finally, *c* is a fitting constant.

**Figure 6:**
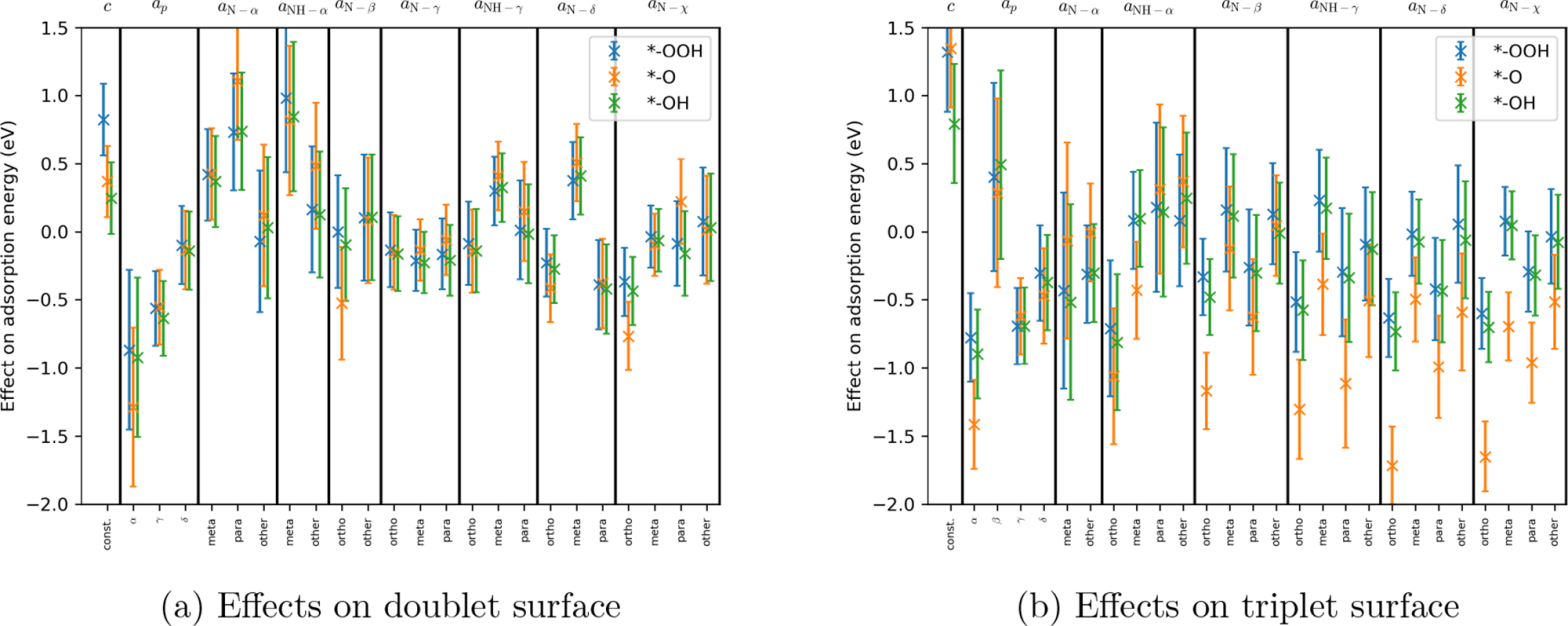
Effect of the absolute position of the active site on graphene and the type and relative position of N’s on the adsorption energies based on fitted parameters of eq 13.

We separated the data according to the multiplicity of the graphene sheet in line with our findings presented in the previous section. Since we did not have data for every position, in a few cases, we could not determine the effect of an active site or N position. The fitting was also limited to the reaction sites where the *–O bond has the most common bond order, 1.5–1.6, and reaction sites with double or epoxy bonds were excluded. For proper fitting, the effect of position χ, *a*_χ_, is considered as the baseline and set to 0. The results of the least-squares fitting with 95% confidence intervals for doublet and triplet surfaces can be seen in Figure 6.

First, let us compare how the effects differ for the three adsorbates. On both types of surfaces, the effects on the adsorption energies of OOH and OH are fairly similar. This is not surprising in light of the linear relationship between them. On doublet surfaces, the trends are also similar for Δ*G*_O_ as for Δ*G*_OOH/OH_, with the exception of *a*_α_, *a*_N-β-ortho_, *a*_N-χ-ortho_, *a*_N-α-para_, *a*_NH-α-other_, and *a*_N-χ-para_. The *a*_α_, *a*_N-β-ortho_, and *a*_N-χ-ortho_ values show a stronger connection compared to OOH and OH, while *a*_N-α-para_, *a*_NH-α-other_, and *a*_N-χ-para_ indicate a weaker interaction. Most of the investigated effects on the O adsorption energies on triplet surfaces are more negative, i.e., the interaction is stronger compared to the effects on Δ*G*_OOH/OH_, except for *a*_β_, *a*_γ_, *a*_δ_, *a*_NH-α-para_, *a*_NH-α-other_, and *a*_N-β-para_.

Second, let us assess the effects of the absolute positions and the positions of the N’s. On both surfaces, the α position strengthens the connection between the species and the surface whereas the β position does not support it. γ and δ have a smaller effect on the adsorption energies than α. On doublet surfaces, N-α and NH-α reduce the chance of adsorption, especially N-α-para and NH-α-meta. N-β, N-γ, and N-χ have a fairly neutral effect on the adsorption energies on a doublet surface, except N-χ-ortho, which promotes the adsorption. In the cases of NH-γ and N-δ, a similar behavior can be observed: the meta position weakens the interaction, while the ortho and para positions are neutral or slightly strengthen it. On triplet surfaces, N-α aids the adsorption in both the meta and the other positions (there is no data for the ortho position because it leads to an absolute β position for the reaction site, and in most cases, OOH is not adsorbed there). NH-α in the ortho position also helps the adsorption, but in other positions, its effect is moderate, closer to zero. For N-γ, we have no data for triplet surfaces. The remaining four N positions, N-β, NH-γ, N-δ, and N-χ, have a similar effect at a particular relative position, ortho > para > other ≳ meta, regarding the effect on the adsorption energies.

**Figure 7:**
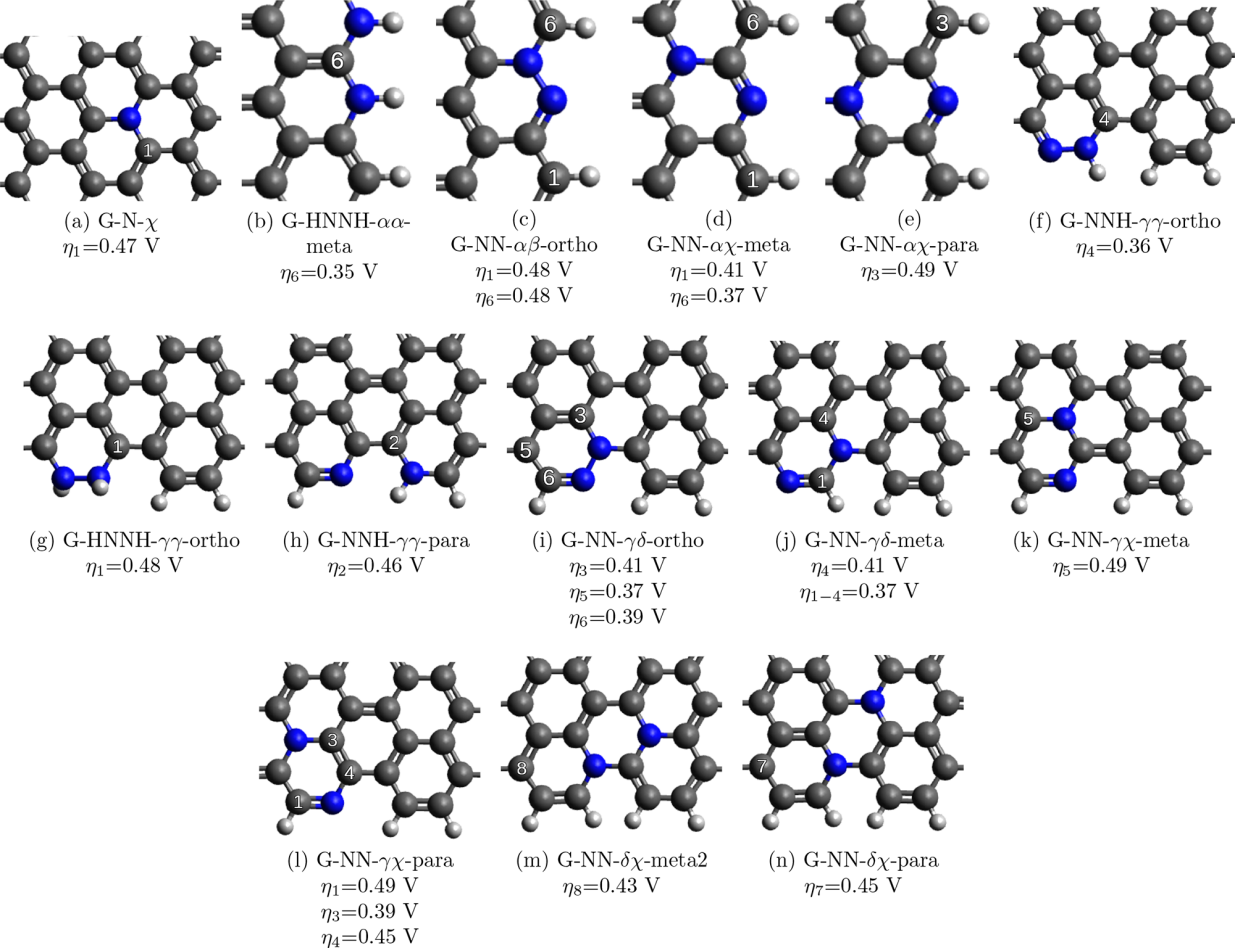
Best active sites with overpotentials under 0.5 V. η_*i*_ refers to the overpotential at position *i* corresponding to the position numbering listed in the SI.

Finally, let us consider the most active reaction sites. These are presented in Figure 7, while the complete list of the energies of the intermediates can be found in Table S1 of the Supporting Information. The results show that considering multiply doped positions, the number of possible active sites is increased, and they are more diverse. On the basis of the best-performing active sites, three strategies can be identified to optimize the graphene surface for the ORR. One way is to enhance the interactions between the oxygen-containing species and the otherwise weakly binding position by putting N’s in the ortho position. The most extreme example is G-HNNH-*αα*-meta-6, where the least binding β position is improved by two NH groups in the ortho position. However, overbinding can occur, like in the case of G-NN-*γχ*-meta-2, where the δ position is slightly more active than the β position and the two ortho N’s have too big of an effect on the adsorption, making OH removal the rate-determining step. Another strategy is to weaken the interaction between the good adsorption positions like α and the intermediates by placing one or two N’s in the meta position, like in the case of G-NN-*αχ*-meta-6. The last strategy, which could be called the moderate way, is to take a moderately good adsorption site (γ, δ, χ) and insert N’s with a moderate effect to compensate for each other for balanced adsorption energies leading to low overpotentials. As we can see, the doped graphene surface contains a number of diverse active centers which differs considerably. However, we can always find active sites where the adsorption of OH and OOH is balanced, leading to minimal overpotential.

These results also resolve the contradiction in the literature about which is the most active center on N-doped graphene by showing that any part of the graphene can have a catalytic effect for the ORR with the appropriate configuration of doping.

### Dissociative Route

The ORR is also possible through the dissociative route over two C atoms if there is a N between them. Considering the two active sites and the possible intermediates, six different pathways are possible (see SI), but the reaction usually occurs as
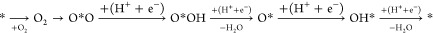
14This pathway could go through the HO–*–OH intermediate; however, in most cases, it is not favored according to our calculations. Due to the variance in O binding and the two active sites, we could not find such relations as for the associative route. Also, we did not attempt to apply such a simple model as for the associative pathway. If the ORR follows the thermodynamically preferred route, where the most stable intermediate is formed in each step, then it is clear that this route is much less effective regarding the highest Gibbs free energy change, which is under 0.5 eV only for one of the sites, G-NN-*γδ*-meta-(1–4) with 0.37 eV. In comparison, the associative pathway had 20 active sites with an overpotential lower than 0.5 V.

We found that either the O_2_ adsorption or the reduction of the second O atom to H_2_O is the rate-determining step on the dissociative route. Even if it is not an efficient route, it should be noted that too strong O_2_ adsorption can initiate this pathway, but then it gets stuck at the last two reduction steps, deactivating the catalytic sites.

However, if intermediates with higher energy can form during the ORR like HO–*–OH or the other O is reduced during the first reduction step, it is possible to get a lower overpotential than for the thermodynamically preferred route. Even the theoretical limit of the associative pathway can be surpassed, G-NN-*γχ*-para-(2-A) and G-NN-*γχ*-meta2-(A-3) have routes with Δ*G*_max_ values of 0.19 and 0.27 eV, respectively. For the energy diagram of the possible routes for G-N N*γχ*-para-(2-A), see Figure S1. These results show the possibility of creating more efficient catalysts for the ORR, but the other available reaction pathways should be altered to reach that goal. Further details and the full list of intermediate energies for each possible dissociative route can be found in Table S2.

### Applicability of the Results

Finally, we discuss several important points which can be decisive for the practical utilization of our results, such as the synthesizability, stability, and conductivity of the modified graphenes. The desired doped graphenes can be fabricated in several ways. A recent review^80^ collected the methods with which one can create such surfaces with the N’s in the desired positions. Doping of pristine H-terminated graphene can be implemented with nitrogen or ammonia plasma. Also, doping can occur in situ with chemical vapor decomposition. These methods can be tuned for specific needs.

The stability of these surfaces is also important from a practical point of view. The thermodynamic stability can be quantified by the Gibbs free energy of the following reaction with Δ_*f*_*G*(C) = 671.244 kJ/mol from the NIST-JANAF tables^81^
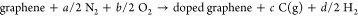


The active surfaces shown in Figure 7 are slightly less stable than pristine graphene with the Gibbs free energy of reaction ranging from −34 to 285 kJ/mol. However, none of the aforementioned surfaces is as outstandingly unstable as, for example, the most unstable one with a reaction free energy of 390 kJ/mol. It must be noted that thermodynamic stability alone cannot predict the real stability of the materials. A study of Chaban and Prezhdo^82^ showed that at high temperatures the N–N bonds are unstable and lead to the degradation of the catalyst. Therefore, formation of neighboring N’s should be avoided during preparation. This means that 4 of the best-performing surfaces, G-NN-*αβ*-ortho, G-NNH-*γγ*-ortho, G-HNNH-*γγ*-ortho, and G-NN-*γδ*-ortho, are probably not practical.

The conductivity of the catalyst is another crucial property which should be discussed. We could estimate the conductivity from the HOMO–LUMO gaps, but they would be far from the band gaps.^83^ However, a recent study of Kim et al.^15^ shows that the conductivity is a function of the ratio of the sp^2^ carbons. Therefore, conductivity depends much more on the density of the modifications rather than the specific doping.

## Conclusions

The oxygen reduction reaction was investigated on several different configurations of graphene surfaces containing one or two N atoms using density functional theory and a computational hydrogen electrode model, focusing on the interactions of N atoms. For the first reduction step, we found that even if the O_2_ adsorption is unlikely, the coupled adsorption of O_2_ and H^+^ to form HO_2_^+^* is a viable path to start the ORR.

The energies of the intermediates of the associative pathway (OOH*, O*, and OH*) were determined, and we found that the scaling relations are also true for the adsorption energies of oxygenated species on graphene surfaces, i.e., there is a linear relationship between the adsorption energies of the three species, and the slope is related to the ratio of bond orders between the surface and the species. Because of the various and unusual bondings of the O atom to the surface, the linear relation is not as descriptive, but differentiating by the surface multiplicity and taking the most common bond, the one of order ≈ 1.5, the scaling relation holds. This unusual bond originates from the interaction of the nonbonding p orbitals of O and the C atoms. The relationship between the adsorption energies of OOH* and OH* leads to a minimal overpotential of 0.33 V. Therefore the adsorption of OH and OOH should be balanced, and one should also consider the potential overbinding of O leading to higher overpotentials.

The relationship of the activity of the sites with their absolute position and the type and relative position of N’s nearby was studied systematically. A simple linear model was fitted to estimate the effect of the different positions on the energetics. On the basis of this model and the active sites with the lowest overpotential, there is no clear blueprint to design the “best” N-doped graphene catalyst but there are several options. One can take a good adsorption site, like the α position, and balance the adsorption energies with N’s in the meta positions. Another viable technique is to take a position where the adsorption is bad, that is, β, and improve the adsorption by adding N’s in the ortho (or para) positions. Between the two extremes, there are a lot of options by adding N’s near moderately good adsorption sites, γ, δ, and χ, to balance out the adsorption energies of OH and OOH. The multiple options for the construction of active sites also explain the seemingly contradicting results in literature. As Figure 7 shows, the graphitic-N and N’s at the ZZ and AC edges in the proper configuration can all create active sites for the ORR.

The dissociative route was also studied, but due to the fact that the ORR occurs on two carbon atoms at the same time and the O binding is diverse, we could not find such relations as for the associative route. However, the results show that only one arrangement provides an overpotential under 0.5 V (0.37 V); therefore, this pathway is not practical. In addition, strong O_2_ adsorption can block the reaction sites and hinder the ORR. However, routes not preferred thermodynamically showed the possibility of breaking the theoretical limit of the overpotential of the associative pathway.

Our findings can contribute to the understanding of the oxygen reduction reaction in polymer electrolyte fuel cells using N-doped graphene and to the design of more efficient electrode materials.

## Supporting Information Available

Energies of the studied systems for the associative and dissociative routes; relevant p orbitals of O to understand the unusual *–O bond (ZIP)
