# Supplementary material for: Oxygen Reduction Reaction on N-Doped Graphene: Effect of Positions and Scaling Relations of Adsorption Energies
Source: J Phys Chem C Nanomater Interfaces. 2021 Apr 20;125(16):8551–61. doi: 10.1021/acs.jpcc.0c11340 (PMC8161692; doi:10.1021/acs.jpcc.0c11340)
Supplement: Supplementary file 1 — jp0c11340_si_002.zip [file jp0c11340_si_002.zip › SI.pdf]

Supporting Information for the Article "Oxygen Reduction Reaction on N-doped Graphene: Effect of Positions and Scaling Relations of Adsorption Energies"

Ádám Ganyecz* and Mihály Kállay

Department of Physical Chemistry and Materials Science, Budapest University of Technology and Economics, Budapest P.O.Box 91, H-1521 Hungary

E-mail: ganyecz.adam@mail.bme.hu

The Supporting Information includes this document and an attached spreadsheet. The spreadsheet contains the relevant energies of the intermediates of the associative and dissociative pathways of the oxygen reduction reaction in Tables S1 and S2, respectively.

Associative pathway

In Table S1, column A presents the images of the surfaces, while column B its names. In column C, the position refers to the image in column A. Multiplicity in column D is the multiplicity of the clean surface. Column E contains additional notes about the *–O bond if it is different from the usual bond, i.e., epoxide, double bond, or a H is also transferred to the O (OH-swap). Columns G-J show the G_{298} energies in Hartree at 0 V vs. SHE and pH=1. "NA" means that no adsorption occurs, and thus further intermediates were not calculated. Columns L-P present the relative ΔG energies at 0 V vs. SHE according to eqs. 5-7. In most cases, if $\Delta G_{\mathrm{OOH}*}$ were larger than 0 eV, the further intermediates were not considered due to the high energy requirement at 1.23 V vs. SHE. Columns R-V display the relative ΔG energies at 1.23 V vs. SHE. Columns X-AB show the ΔG_i energy steps required at 1.23 V vs. SHE according to eqs. 8-11 along with $\Delta G_{\max} = \max\{\Delta G_1, \Delta G_2, \Delta G_3, \Delta G_4\}$.

Dissociative pathway

During the investigation of the dissociative pathway, the following 6 routes were considered:

- A: * → O_2* → O*O → O*OH → O* → HO* → *
- B: * → O_2* → O*O → HO*O → *O → *OH → *
- C: * → O_2* → O*O → O*OH → HO*OH → HO* → *
- D: * → O_2* → O*O → O*OH → HO*OH → *OH → *
- E: * → O_2* → O*O → HO*O → HO*OH → HO* → *
- F: * → O_2* → O*O → HO*O → HO*OH → *OH → *

In Table S2, columns A-D are the same as in Table S1. Here, position refers to the two C atoms to which the two O atoms are connected. The additional positions which were not studied on the associative pathway are labeled as "A" and "B". The two reaction sites of the dissociative pathway are distinguished. In notations O*O, O*OH, HO*O, HO*OH, O*, *O, HO*, and *OH, the species before the * mark refers to the corresponding species in the first position, while the species after the * mark reflects that the species is in the second position. For example, at position 3-6, the O*OH notation means that at carbon atom 3, there is an O, while at atom 6, there is an OH. Columns G-P show the G_{298} energies in Hartree at 0 V vs. SHE and pH=1, "NA" means that no adsorption occurs, and thus further intermediates were not calculated. Columns S-AC and AE-AO present the relative ΔG energies at 0 V and 1.23 V vs. SHE in a manner analogous to Table S1. Columns AQ-AV summarize the ΔG_{max} values for each pathway, while Column AW shows the thermodynamically preferred route. Columns AY-BE, BG-BM, BO-BU, BW-CC, CE-CK, and CM-CS display in detail the ΔG_i energy steps required at 1.23 V vs. SHE along with the energy needed for the proper O_2 adsorption (ΔG_{ads}) and O–O bond dissociation (ΔG_{diss}) for each possible route.

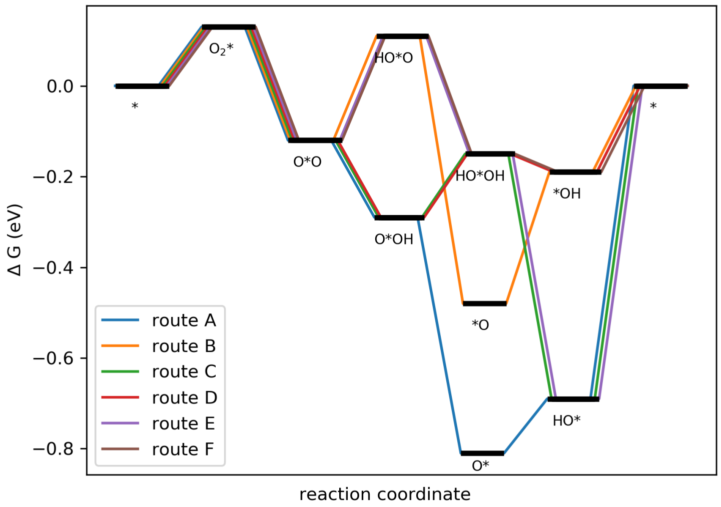

Figure S1: Possible routes for G-NN-$\gamma\chi$-para-(2-A) at 1.23 V vs SHE.

As an example, Figure S1 shows all the possible routes for G-NN-$\gamma\chi$-para-(2-A) at 1.23 V vs SHE. It can be seen that thermodynamically, route A is preferred because it is easier to form O*OH than HO*O as well as O* than HO*OH. However, it leads to an overpotential of 0.69 V. If we could prevent the formation of HO*, then it would be possible to reach overpotentials under 0.3 V with routes B, D, and F.

The nature of the *–O bond

In Figure S2, the relevant p orbitals of O are shown for the three intermediates of G-HNNH-$\alpha\alpha$-meta-6. The canonical orbitals were localized using the Pipek–Mezey method with Mulliken populations[1] with the aid of Multiwfn.[2] The contributions of different atoms to the orbitals were determined with the Hirshfeld method.[3,4] The orbitals were visualized with iqmol[5] using an isovalue of 0.2. Looking at the localized orbitals of O*, it can be realized that the non-bonding p orbitals of O interact with the C atom giving a small π bond character to the p orbitals, which explains the non-integer part of the bond order. In the cases of OOH* and OH*, this interaction does not exist, the non-bonding p orbitals of O are undisturbed.

Surface	Orbitals	
OOH*	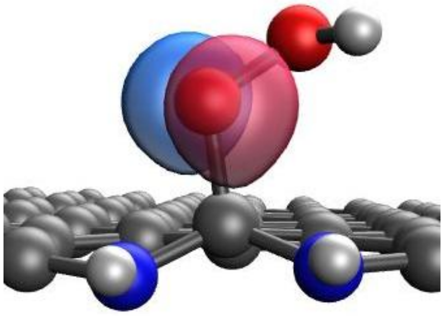<br>86 % O, 5 % C	
O*	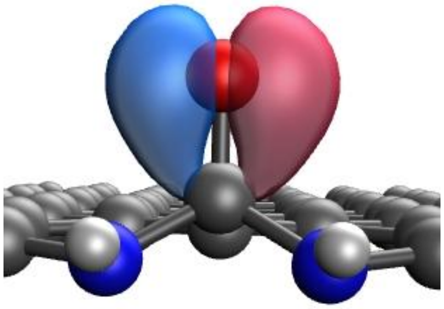<br>83 % O, 11 % C	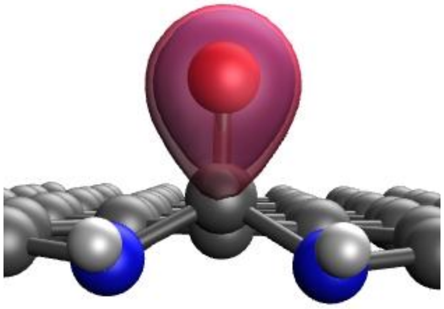<br>82 % O, 10 % C
OH*	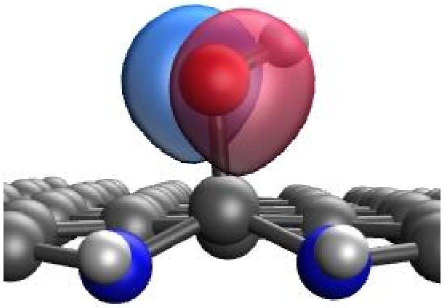<br>84 % O, 6 % C, 5 % H	

Figure S2: Relevant p orbitals of the three intermediates of G-HNNH-$\alpha\alpha$-meta-6. Only the α orbitals are presented because the β orbitals are essentially same. The major contributors to the orbitals are also shown based on the Hirshfeld method.

References

(1) Pipek, J.; Mezey, P. G. A fast intrinsic localization procedure applicable for abinitio and semiempirical linear combination of atomic orbital wave functions. *J. Chem. Phys.* **1989**, *90*, 4916–4926.

(2) Lu, T.; Chen, F. Multiwfn: a multifunctional wavefunction analyzer. *J. Comp. Chem.* **2012**, *33*, 580–592.

(3) Tian, L.; Feiwu, C. Calculation of molecular orbital composition. *Acta Chim. Sinica* **2011**, *69*, 2393–2406.

(4) Hirshfeld, F. L. Bonded-atom fragments for describing molecular charge densities. *Theor. Chim. Acta* **1977**, *44*, 129–138.

(5) Gilbert, A. IQmol. 2019.
